# High-Throughput Sequencing to Detect Novel Likely Gene-Disrupting Variants in Pathogenesis of Sporadic Brain Arteriovenous Malformations

**DOI:** 10.3389/fgene.2020.00146

**Published:** 2020-02-28

**Authors:** Concetta Scimone, Luigi Donato, Concetta Alafaci, Francesca Granata, Carmela Rinaldi, Marcello Longo, Rosalia D’Angelo, Antonina Sidoti

**Affiliations:** ^1^ Department of Biomedical and Dental Science and of Morphological and Functional Images, University of Messina, Messina, Italy; ^2^ Department of Vanguard Medicine and Therapies, Biomolecular Strategies and Neuroscience, I.E.ME.S.T., Palermo, Italy

**Keywords:** brain arteriovenous malformations, pathogenic variants, exome, molecular signaling, vascular differentiation

## Abstract

Molecular signaling that leads to brain arteriovenous malformation (bAVM) is to date elusive and this is firstly due to the low frequency of familial cases. Conversely, sporadic bAVM is the most diffuse condition and represents the main source to characterize the genetic basis of the disease. Several studies were conducted in order to detect both germ-line and somatic mutations linked to bAVM development and, in this context, next generation sequencing technologies offer a pivotal resource for the amount of outputted information. We performed whole exome sequencing on a young boy affected by sporadic bAVM. Paired-end sequencing was conducted on an Illumina platform and filtered variants were validated by Sanger sequencing. We detected 20 likely gene-disrupting variants affecting as many loci. Of these variants, 11 are inherited novel variants and one is a *de novo* nonsense variant, affecting *STK4* gene. Moreover, we also considered rare known variants affecting loci involved in vascular differentiation. In order to explain their possible involvement in bAVM pathogenesis, we analyzed molecular networks at Cytoscape platform. In this study we focus on some genetic point variations detected in a child affected by bAVM. Therefore, we suggest these novel affected loci as prioritized for further investigation on pathogenesis of bAVM lesions.

## Introduction

Brain arteriovenous malformations (bAVM, OMIM #108010) are congenital malformations affecting microvessels. The absence of the capillary bed is peculiar of these lesions resulting in pathological direct shunt from arterioles to venules. Feeding arteries continue in an undifferentiated arteriovenous nidus that resolves in a single or more draining veins. At nidus, the vessels are enlarged and susceptible to rupture. Arterial and venous circulation are mixed within these lesions, therefore blood perfuses with a high pressure, increasing bleeding risk (Goldberg et al., 2018). Intracerebral hemorrhage (ICH) is the most common clinical manifestation appearing in about 50% of patients. The incidence of bAVM is approximately 0.01% and usually it develops sporadically (Osbun et al., 2017). Multiple lesions occur coexisting together with other vascular malformation syndromes, like hereditary hemorrhagic telangiectasia (HHT). HHT patients show multiple AVM affecting visceral organs and multiple telangiectasia at mucosal tissues. Mutations at genes involved in the TGF-βII transduction pathway cause different HHT phenotypes (Pawlikowska et al., 2018), (Karlsson and Cherif, 2018). Familial bAVM without HHT is extremely rare and only about 30 affected families were reported (van Beijnum et al., 2007). Although genetic causes are not yet elucidated, pedigree analysis confirmed an autosomal dominant pattern of inheritance with incomplete penetrance and variable expressivity. Three linkage studies were performed on 15 affected families and several chromosomal loci were mapped despite the exact genes are still unidentified (Oikawa et al., 2010). Mutational analyses were performed on ephrin family genes mapping on these regions but without success (Inoue et al., 2007). Recently, a novel missense mutation in *ACVRL1* gene was reported as segregating within a family affected by hereditary bAVM without HHT (Yılmaz et al., 2017).

Expression studies performed on human lesion-derived specimens revealed alterations at several pathways controlling angiogenesis and angio-architecture maintenance, highlighting that lesions can arise due to impaired expression of ephrin proteins during early angiogenesis, following Hedgehog (Hh)-VEGF-Notch signaling activation (Lawson et al., 2002). This results in *EFNB2* down-expression and loss of vein differentiation. Conversely, venous markers *FLT4* and *EPHB4* result expressed in arteries as consequence of notch signaling down-regulation (Lawson et al., 2001).

Next generation sequencing (NGS) technology, in the last years, is allowing to rapidly increase information regarding disease-causing loci. Here we present results of a whole exome sequencing (WES) performed on a young patient affected by sporadic bAVM. With support of bioinformatic tools and network analysis, likely gene-disrupting (LGD) variants at 20 loci, potentially linked to bAVM development, were detected.

## Methods

### Whole Exome Sequencing and Bioinformatic Analysis Pipeline

WES analysis was performed on a Caucasian child who manifested ICH due to a single AVM at the right cerebellar hemisphere. The lesion was totally resected following two surgeries and, to date, no recidivism is reported. However, he recently developed varicocele. His parents were unaffected despite the mother and the maternal grandmother showed varicose veins. The patient was classified as sporadic.

WES analysis was carried on DNA purified from whole peripheral blood by the QIAamp DNA Mini Kit (Qiagen). The extraction yield was assessed at NanoDrop spectrophotometer (Thermo Scientific). Exome capturing was performed by the SureSelectXT Human All Exon V6” (Agilent Genomics) kit. Library was set up in order to generate 100 bp length reads. Paired-ends sequencing was run on a Illumina HiSeq 2500 System.

The generated reads were subject to bioinformatic analysis, as described: quality check was performed by FastQC_Version_0.11.7 (http://www.bioinformatics.babraham.ac.uk/projects/fastqc) tool. Reads presenting a Phred score ≥ 28 were selected, trimmed, and aligned to the reference human genome GRCh37 (hg19) by the Burrows-Wheeler Aligner (BWA) (Li and Durbin, 2009). Duplicate reads were removed by Picard toolkit Version_2.18.23 (Picard Toolkit.” 2019. Broad Institute, GitHub Repository. http://broadinstitute.github.io/picard/; Broad Institute). Genome Analysis Toolkit Version_4.1.3.0 (GATK) (https://software.broadinstitute.org/gatk/), ANNOVAR Version_2018Apr16 (Wang et al., 2010), and SnpEff Version_4.3T (Cingolani et al., 2012) were used for variant calling and variant annotation, respectively. Frequencies of detected variants were defined by the 1000 Genomes database.

### Selection of Likely Gene-Disrupting Variants and Sanger Validation

LGD variants potentially involved in bAVM pathogenesis were strictly selected as follows. Firstly, all variants not reported in public human genome mutation database (HGMD^®^) or in SNP collection databases as dbSNP (https://www.ncbi.nlm.nih.gov/snp/), ExAC (http://exac.broadinstitute.org/), and Ensembl (https://www.ensembl.org/index.html) were considered. For variants already reported in literature, the first selective criterion was the minor allele frequency (MAF) reported in the 1000 Genome Project database and the cut-off value MAF < 0.01 (1%) was selected. Variants presenting MAF < 0.01% were further classified according to their functional class and missense, nonsense, frameshift, and splice site variants were considered. Within each group, we selected all those resulted potentially pathogenic at LRT (http://www.genetics.wustl.edu/jflab/lrt_query.html) (Chun et al., 2009 **), MutationTaster (http://www.mutationtaster.org) (Schwarz et al., 2014), and SIFT 4G (10.1038/nprot.2015.123) (Vaser et al., 2016) prediction tools.

For each functional class, variants showing low depth value were excluded. Subsequently, gene prioritization was established by enrichment analysis performed on Gene Ontology database, in relation to Biological Process annotations and Reactome pathways. Filtered LGD variants were validated by Sanger sequencing by using the BigDyeTerminator^©^ v3.1 Cycle Sequencing Kit and run on a 3500 Genetic Analyzer (Applied Biosystems, Thermo Fisher Scientific), after amplification. Primer pairs and polymerase chain reaction conditions are provided as **Supplementary Materials (SM1)**. Finally, physical and predicted interactions, co-expression, co-localization, and shared domain patterns of LGD gene sets were obtained by GeneMania Version_3.5. plugin 1 (Montojo et al., 2010) of Cytoscape Version_3.6.0 platform.

### Screening of the Relatives

In order to detect possible *de novo* mutations and to establish segregation of selected variants within the family, the parents of the proband were genotyped by Sanger sequencing.

### Allele Frequency Estimation

The frequencies of the novel variants were estimated in healthy population by restriction fragment length polymorphism (RFLP) analysis and Sanger sequencing. Enzymatic digestion was applied for variants in *CLCN2*, *EFNA4*, *IGFBP7*, *TNXB*, *L2HGHD* genes. Five units of specific enzyme were used to digest 0.5 µg of DNA. The restriction enzymes and the obtained fragments are listed in SM1. Variants detected in *NAXE*, *TTLL11*, *TRMT10B*, *AOC3*, *STK4*, *SLIT2*, and *FLRT3* genes do not affect any restriction site, therefore these were screened by Sanger sequencing, as previously described.

Control group was made up by 106 and 94 Caucasian healthy males and females respectively, unrelated and randomly selected. The absence of lesions was confirmed by MRI investigation.

### RNA Extraction, Quantitative Real-Time PCR, and Statistical Analysis

White blood cells (WBCs) were isolated from peripheral blood by Ficoll (Lympholyte^®^ Cell Separation Media, Cedarlane). Total RNA was purified using TRIzol^®^ reagent (Thermo Fisher Scientific), following manufacturer protocol. For each reaction, 1 µg of total RNA was retro-transcribed using the GeneAmp RNA PCR Core Kit^®^ (Thermo Fisher Scientific) and applying the two-step protocol, as suggested in the datasheet. RT-PCR was conducted to evaluate the nonsense-mediated decay (NMD) as consequence of the nonsense mutation detected in *STK4* gene. *STK4* expression level in lymphocytes of the patient was compared with that observed in the negative control. The 2-years younger brother of the proband was considered as negative control. The absence of any *STK4* nonsense mutation in the negative control was proven by Sanger sequencing. Equimolar quantities of sample/control complementary DNA (cDNA) were used for specific *STK4* coding sequence amplification using the following primer pair: 5’-GAATACAGTGATAGGAACAC–3’ and 3’-CTCTTTACAAGACACTGTT-5’. The TaqMan^®^ probe was designed downstream to the mutation site, in order to cover the exon-exon boundary. The probe had the following sequence 5’-VIC-CATCCAATGAGGGCAATCTTCATG–TAMRA-3’. For data normalization, human *GAPDH* housekeeping gene was used as endogenous reference. The probe for *GAPDH* was labeled with FAM as fluorophore and TAMRA as quencher. Multiplex TaqMan reactions were performed using the TaqMan^®^ Master Mix (Thermo Fisher Scientific). For each reaction, 10 ng of cDNA were added to 3 µM of each primer, 5 µM of each probe and to the Master Mix. UltraPure™ DNase/RNase-Free Distilled Water was added up to reach 20 µl of total reaction volume. Quantitative RT-PCR reactions were conducted on a 7500 Real-Time PCR System (Applied Biosystems). RNA purification and RT-PCR reactions were trice replicated. Before multiplex RT-PCR reactions, singleplex reactions were conducted in order to evaluate the single threshold cycle (Ct) values of each amplicon. Different abundance of the *STK4* cDNA was evaluated between the sample and the negative control. Relative quantification was determined by the 2-^ΔΔCt^ method and was normalized against the expression of the human *GAPDH* gene. All data analyses were performed using the IBM SPSS 24 software for Macintosh (IBM Corp. Released 2013. IBM SPSS Statistics for Macintosh, Armonk, NY). A one-way analysis of variance (one-way ANOVA) was performed to compare between the sample groups. All p-values were Bonferroni’s corrected and considered significant if p < 0.05.

### Protein Extraction and Western Blot Analysis

Proteins were extracted from WBCs, previously isolated from whole peripheral blood by Ficoll (Lympholyte^®^ Cell Separation Media, Cedarlane). Radioimmunoprecipitation assay (RIPA) buffer supplied with protease inhibitor mix was used. Also in this case, the negative control considered was the younger brother of the proband and the samples were in parallel processed. Colorimetric protein quantification was performed by the Bradford protein assay. The protein amount of 30 µg was denatured, separated on a sodium dodecyl sulfate (SDS) (10%) 15% polyacrylamide gel and then blotted on a Immun-Blot^®^ PVDF Membrane (Bio-Rad) by running for 2 h at 80 mA with transfer buffer (39 mM glycine, 48 mM Tris, 20% methanol) at 4°C. Membrane was stained in TBS 0.1%-Tween and blocked with 5% non-fat dry milk for 1 h at room temperature and then thrice washed in TBS–0.1% Tween. Overnight incubation with the primary anti-STK4 antibody (Genetex International Corporation, Hsinchu City, Taiwan, R.O.C.) was conducted in TBS-0.1%-Tween. Primary anti-STK4 antibody was 1:2,000 diluted. This step was followed by three washings in TBS–0.1% Tween and by the incubation with the secondary antibody peroxidase-conjugated goat anti-rabbit immunoglobulin G (Pierce, Rockford, IL, USA). Secondary antibody was diluted 1:20,000 and incubation was performed at room temperature for 1 h. In parallel, membrane for the β-actin immunodetection was processed for data normalization. Images were developed by the enhanced chemiluminescence system (ECL) (Amersham, Little Chalfont, UK) and digitally acquired (DiGit Blot Scanner with Image Studio 4.0 software, LI-Cor, Lincoln, NE, USA). Densitometric analysis was performed in order to calculate the STK4 protein amount of the sample compared to the control by Gel-Pro Analyzer v.4 software.

### Ethical Statement

The study was approved by the local Ethical Committee (A.O.U. “G. Martino,” Messina, Italy) and it is compliant with Helsinki declaration.

All recruited subjects were fully informed ad written consent was obtained, also for the underage subjects.

## Results

### Whole Exome Sequencing and Bioinformatic Analysis

Sequencing run generated 86,816,620 reads. Phred quality score greater than 30 (Phred > 30) resulted for 92.83% of total reads. After duplicates discard, 69,592,810 were filtered and 99.09% mapped with hg19 RefSeq. Among these, 82.92% were on-target reads. Main reports of the sequence run, variant calling, and annotation results are provided as **Supplementary Materials (SM2)**.

### Selection of Likely Gene-Disrupting Variants and Sanger Validation

With exception of deep intronic variants, analysis outputted 63,171 variants. Of these, only 11,205 showed MAF < 0.01% and were clustered by functional class.

**Figure 1:**
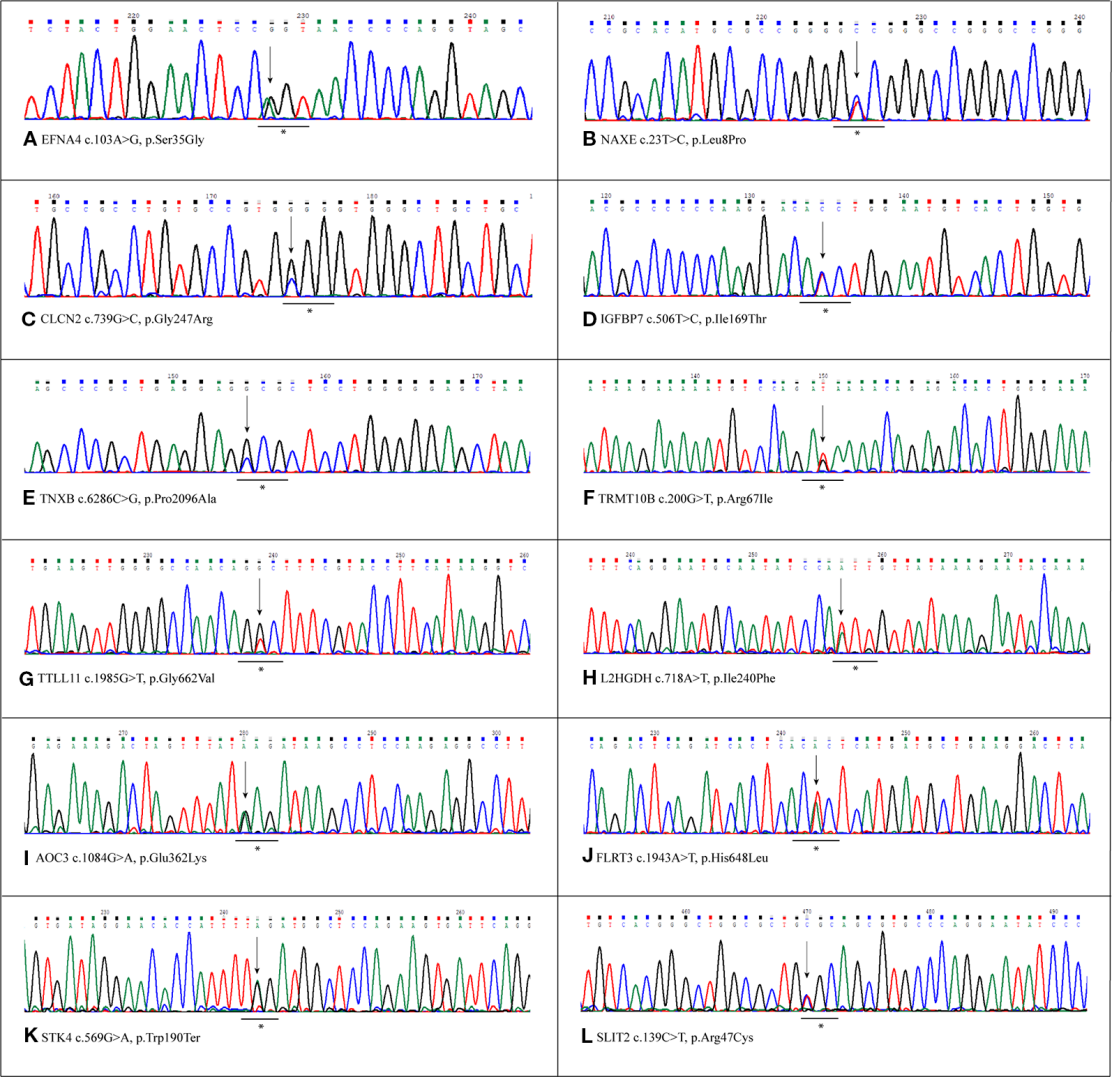
Sanger validation of novel variants. Electropherograms show the novel variants detected by whole exome sequencing (WES) and confirmed by Sanger sequencing. Each panel refers to the single variant described at the bottom of the same panel. The underlined asterisked triplets indicate the codons affected by mutations. All variants were detected in heterozygous condition. **(A)** Variant detected in EFNA4 gene, c.103A>G, p.Ser35Gly. **(B)** Variant detected in NAXe gene,c.23T>C, p.Leu8Pro. **(C)** Variant detected in CLCN2 gene, c.739C>G,p.Gly247Arg. **(D)** Variant detected in IGFBP7 gene, c.506T>C, p.Ile169Thr. **(E)** Variant detected in TNXB gene, c.286C>G, p.Pro2096Ala **(F)** Variant detected in TRMT10B gene, c.200G>T,p.Arg67Ile. **(G)** Variant detected in TTLL11 gene, c.1985G>T, p.Gly662Val. **(H)** Variant detected in L2HGDH gene, c.718A>T, p.Ile240Phe. **(I)** Variant detected in AOC3 gene, c.1084G>A, p.Glu362Lys **(J)** Variant detected in FLRT3 gene, c.1943A>T, p.His648Leu. **(K)** Variant detected in STK4 gene, c.569G>A, p.Trp190Ter. **(L)** Variant detected in SLIT2 gene, c.139C>T,p.Arg47Cys.

Variants resulted potentially damaging by the three prediction tools were selected. The Gene Ontology annotation of biological processes and the reactome pathway analysis allowed to totally select 20 likely gene-disrupting (LGD) variants. In particular, eight are known single nucleotide variants (SNV) while 12 are novel, not yet reported in HGMD^®^ or other variant collection databases. All selected variants were validated and confirmed by Sanger sequencing. **Table 1** lists the 20 LGD variants selected as potentially involved in bAVM onset. Of these, 17 are missense, 2 are splicing variants, and only 1 is a nonsense variant (c.569G > A, p.Trp190Ter) affecting *STK4* gene. Electropherograms were obtained in order to validate novel variants and are shown in **Figure 1**.

**Table 1:** Likely gene-disrupting (LGD) variants selected as potentially involved in arteriovenous malformation (AVM) onset.

Locus	Gene (Entrez Gene ID)	Functional class	Variant	rs ID	MAF 1000 genomes	GO Biological process—reactome pathway	Pattern of inheritance
1q21.1	NBPF10 (100132406)	Splicing variant (intronic)	c.1308-4G > A	rs4110417	Not reported	R-HSA-9006936.3 Signaling by TGF-beta family members	Maternal
						R-HSA-2173788.1 Downregulation of TGF-beta receptor signaling	
						R-HSA-2173789.1 TGF-beta receptor signaling activates SMADs	
1q21.3	EFNA4 (1945)	Missense	c.103A > G, p.Ser35Gly	Novel	Not reported	GO:0048013 ephrin receptor signaling pathway (GO:0048013)	Maternal
1q25.2	ABL2 (27)	Missense	c.2789A > G, p.Lys930Arg	rs17277288	0.005	GO:2000352 negative regulation of endothelial cell apoptotic process	Paternal
1q22	NAXE (128240)	Missense	c.23T > C, p.Leu8Pro	Novel			Maternal
2q24.3	TTC21B (79809)	Missense	c.179T > A, p.Phe60Tyr	rs371571631	< 0.01	GO:0007224 smoothened signaling pathway	Maternal
3q27.1	CLCN2 (1181)	Missense	c.739G > C, p.Gly247Arg	Novel			Paternal
4q21.21	BMP3 (651)	Missense	c.1204G > C, p.Ala402Pro	rs147182183	9.127e−05	GO:0010862 positive regulation of pathway-restricted SMAD protein phosphorylation	Maternal
						GO:0060395 SMAD protein signal transduction	
4q12	IGFBP7 (3490)	Missense	c.506T > C, p.Ile169Thr	Novel	Not reported	GO:0001569 branching involved in blood vessel morphogenesis	Paternal
						GO:0002040 sprouting angiogenesis	
4p15.31	SLIT2 (9353)	Missense	c.139C > T, p.Arg47Cys	Novel	Not reported	GO:0043116 negative regulation of vascular permeability	Paternal
						GO:0010596 negative regulation of endothelial cell migration	
6q13	CD109 (135228)	Missense	c.1709C > T, p.Pro570Leu	rs41266745	0.0006	GO:0030512 negative regulation of transforming growth factor beta receptor signaling pathway	Paternal
6p21.33-p21.32	TNXB (7148)	Missense	c.6286C > G, p.Pro2096Ala	Novel	Not reported		Maternal
7q11.23	NCF1 (653361)	Missense	c.269G > A, p.Arg90His	rs201802880	0.001	GO:0048010 vascular endothelial growth factor receptor signaling pathway	Paternal (homozygous)
						GO:0002042 cell migration involved in sprouting angiogenesis	
9p13.2	TRMT10B (158234)	Missense	c.200G > T, p.Arg67Ile	Novel	Not reported		Maternal
9q33.2	TTLL11 (158135)	Missense	c.1985G > T, p.Gly662Val	Novel	Not reported		Maternal
14q21.3	L2HGDH (79944)	Missense	c.718A > T, p.Ile240Phe	Novel	Not reported		Paternal
15q24.2	CSPG4 (1464)	Missense	c.3239G > A, p.Arg1080His	rs374794981	8.378e−06	GO:0001525 angiogenesis	Paternal
17q21.31	AOC3 (8639)	Missense	c.1084G > A, p.Glu362Lys	Novel	Not reported		Maternal
18q21.31	NEDD4L (23327)	Splicing variant (intronic)	c.2488-7C > T	rs746481322	< 0.01	R-HSA-201451.4 Signaling by BMP	Maternal
						R-HSA-2173793.2 Transcriptional activity of SMAD2/SMAD3:SMAD4 heterotrimer	
						R-HSA-170834.1 Signaling by TGF-beta Receptor Complex	
						R-HSA-2173788.1 Downregulation of TGF-beta receptor signaling	
						R-HSA-2173795.1 Downregulation of SMAD2/3:SMAD4 transcriptional activity	
						R-HSA-2173789.1 TGF-beta receptor signaling activates SMADs	
20q13.12	STK4 (6789)	Nonsense	c.569G > A, p.Trp190Ter	Novel	Not reported	GO:0001569 branching involved in blood vessel morphogenesis	***De novo***
20p12.1	FLRT3 (23767)	Missense	c.1943A > T, p.His648Leu	Novel	Not reported		Maternal

LGD variants selected as potentially involved in AVM onset. For each affected gene are reported the chromosome locus, the name and the Entrez Gene ID (1^st^, 2^nd^, 3^rd^ columns, respectively). For each variant are reported the mutation class, the position in coding sequence and, for missense variants, the amino acid change. For known single nucleotide variant (SNV) is also indicated the rs ID by which they are named in collection databases. MAF 1000 Genomes refers to minor allele frequency observed and reported in 1000 Genome database for the specific allele. The last 7^th^ column reports the Gene Ontology biological process and the reactome pathways related to angiogenesis regulation and TGF-β transduction signaling, for each selected gene. Inheritance pattern is reported in the last column.

**Figure 2:**
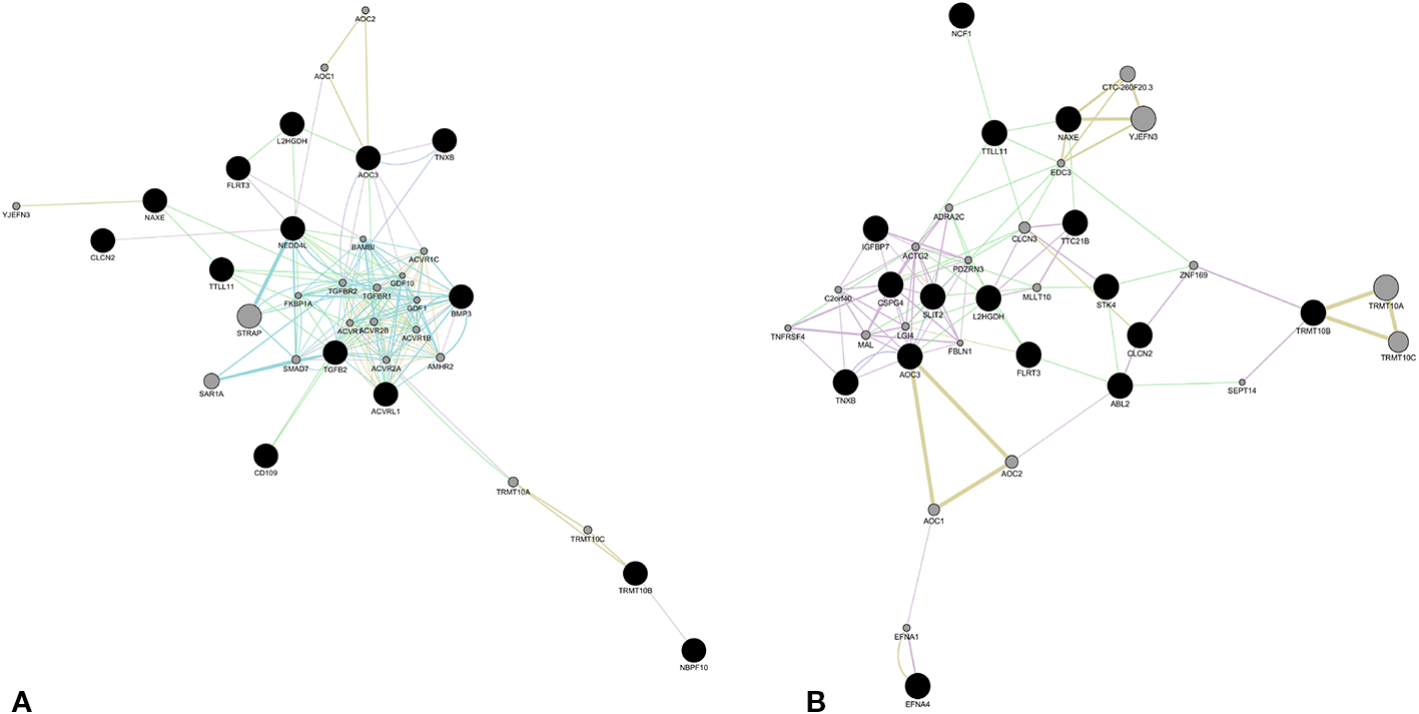
Molecular network obtained by GeneMania plugin (Cytoscape). The network shows patterns of co-expression (violet), co-localization (blue), genetic interaction (green), pathway (light blue), predicted interaction (orange), and shared proteins domains (yellow), obtained from merger of genes affecting by likely gene-disrupting (LGD) variants. The eight novel loci were merged with those involved in TGFβ signaling **(A)** and those involved in angiogenesis and vessel differentiation **(B)**. Color of each edge is in relation of the kind of interaction. Black nodes represent input genes. Gray nodes resulted from analysis. Details about nodes and edges as well as annotations are available in Tables **1–5**, supplied in **Supplementary Materials SM3**.

Regarding affected loci, role of *EFNA4*, *IGFBP7*, *SLIT2*, and *STK4* in vessel maturation was already demonstrated. However there are no pertinent GO annotations for the eight loci *NAXE*, *CLCN2*, *TNXB*, *TRMT10B*, *TTLL11*, *L2HGDH*, *AOC3*, and *FLRT3*. In order to obtain any functional relation among these and the other loci reported in **Table 1**, network analysis by GeneMANIA plugin of Cytoscape platform was performed. We obtained two different networks, showed in **Figure 2**. Details on nodes and edges are provided as **Supplementary Materials (SM3)**. The eight previous novel loci were also given as input together with the other *NBPF10*, *BMP3*, *CD109*, *NEDD4L*, known to be involved in TGF-β transduction pathway, and *TGFB2* and *ACVRL1*, since it resulted mutated in the patient affected by sporadic bAVM (**Figure 2A**). Network showed in **Figure 2B** was obtained giving as input the same eight loci and all those involved in angiogenesis, as *ABL2*, *EFNA4*, *TTC21B*, *IGFBP7*, *SLIT2*, *NCF1*, *CSPG4*, *STK4*.

### Screening of the Relatives

Genotyping of the parents of the young patient revealed that all detected variants were inherited with the exception of the novel nonsense c.569G > A (p.Trp190Ter) affecting *STK4* locus. This variant was not detected in any parents. Patterns of inheritance for each variant are reported in **Table 1**.

### Allele Frequency Estimation

The frequencies of the novel variants were estimated on a cohort of 200 healthy subjects, heterogeneous for sex and age. None of the controls carried the variants, therefore they can be considered mutations (data not shown).

### Real Time-PCR, Gene Expression, and Protein Detection

**Figure 3:**
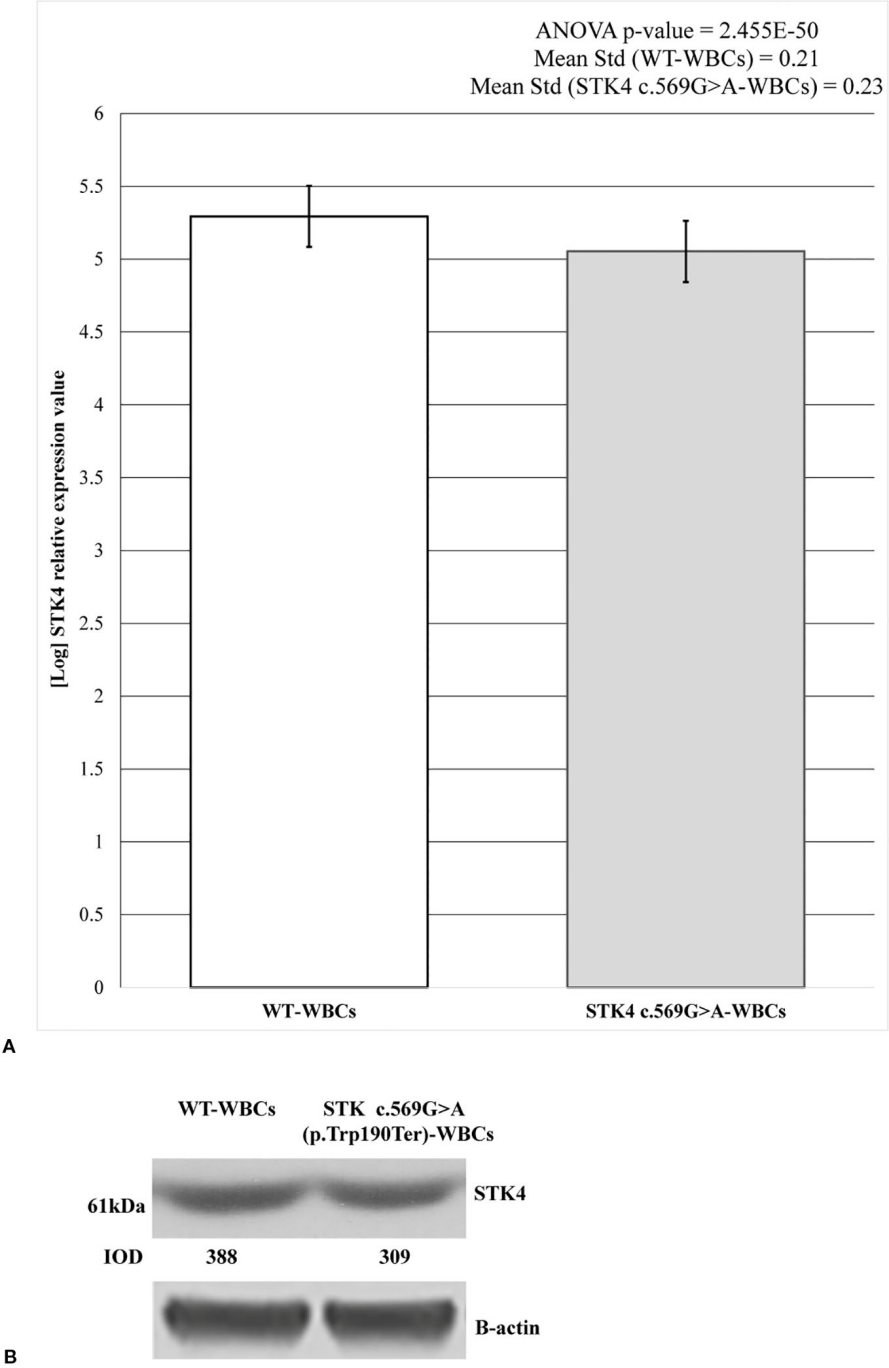
*STK4* expression values and protein detection in control white blood cells (WBCs) and c.569G > A (p.Trp190Ter) WBCs. **(A)** The histogram shows the logarithmic *STK4* relative expression values coming from real-time (RT)-PCR experiment, compared between wild-type and mutated sample groups. As reported, ANOVA test resulted significant (p-value = 2.455E−50). The presence of *STK4* c.569G > A variant determines an expression reduction of about 20%, compared to the wild-type control. Reported values represent the mean of the three replicates. **(B)** Protein detection by western blot analysis showing a reduction of the STK4 protein synthesis in the heterozygous c.569G > A (p.Trp190Ter) mutation carrier. The integrated optical intensity (IOD) values are reported.

*STK4* expression level was measured on both c.569G > A and wild-type WBCs. The mature wild-type messenger RNA (mRNA) consists of 11 exons and the substituted nucleotide is comprised in the 6^th^. It was predicted to create a premature stop codon leading to the synthesis of a 190 amino acid truncated protein against the 487 wild-type one. Therefore the hypothesis of NMD was evaluated as mechanism involved in premature degradation of mRNA molecule transcribed by the mutated allele (Lindeboom et al., 2019). This hypothesis was assayed and confirmed by RT-PCR (**Figure 3A**). The results are reported as logarithmic values and highlight the reduction of about 20% of the *STK4* transcript in the c.596G > A WBCs, compared to the wild-type cells. The ANOVA test was applied to determine statistical significance of the data showing a significative p-value = 2.455E−50. Results are shown as the mean values of the three replicates.

These results were also confirmed by western blot analysis. As predicted, the synthesis of a truncated 21.7 kDa protein results following the c.569G > A (p.Trp190Ter) nonsense mutation (https://web.expasy.org/compute_pi/), compared to the 52.335 kDa wild-type one. The 61 kDa product results from anti-STK4-STK4 complex. As expected, in the c.569G > A heterozygous mutant only the wild-type protein was observed (**Figure 3B**) and this resulted reduced by about the 20% when compared to the wild-type condition, as shown by the densitometric analysis. Taken together, these data highlight that the mutant allele is prematurely degraded probably by the NMD.

## Discussion

Brain AVM is a rare disease usually observed as sporadic condition. Despite the genetic bases of the disease are not yet well elucidated, more evidences indicate possible common pathways as cause of sporadic, hereditary AVM and HHT onset (Walcott et al., 2018). Familial AVM without HHT is an extremely unusual condition, therefore many genetic studies are madeon sporadic patients. Here we reported the main data collected by WES analysis performed on a child affected by sporadic AVM with the aim to increase the very little knowledge regarding genetic and molecular etiology of the disease. Among all detected variants, we firstly focused on the novel ones and then considered those presenting MAF < 0.01 and affecting genes whom GO enrichment revealed any possible involvement in TGF-βII transduction pathway or in angiogenesis regulation. Based on functional class, 1 nonsense, 2 splice affecting, and 17 missense mutations have been considered (**Table 1**). Selected genes were clustered in three groups. The first includes all genes involved in TGF-β/SMAD transduction pathway, as *NBPF10*, *BMP3*, *CD109*, *NEDD4L* genes (Lei et al., 2017; Yamakawa et al., 2018). All detected variants were already reported in ExAC database although no clinical significance was to date assigned.

The second group comprises genes that regulate angiogenetic process as well as arterial and vein differentiation. These genes are *ABL2*, *EFNA4*, *TTC21B*, *IGFBP7*, *SLIT2*, *NCF1*, *CSPG4*, *STK4* (Amyere et al., 2017; Errede et al., 2018; Romano et al., 2018; Tumelty et al., 2018). Among these, *EFNA4*, *IGFBP7*, and *SLIT2* genes are affected by novel missense mutations while *STK4* carries a novel nonsense mutation leading to a 190 amino acid truncated protein against 487 amino acid wild-type one.

In the third cluster are grouped all genes affected by novel variants that showed no annotation with TGF-βII and angiogenetic pathways, and these are *NAXE*, *CLCN2*, *TNXB*, *TRMT10B*, *TTLL11*, *L2HGDH*, *AOC3*, *FLRT3*. Knowledge about these genes are really few and several of them are not yet reported in HGMD database, being not associated to any pathological phenotypes. Briefly, a recent study showed involvement of *NAXE* in vascular remodeling *via* modulation of cholesterol metabolism and γ-secretases, proving the attenuation of Notch signaling and the enhance of angiogenesis following *NAXE* depletion (Mao et al., 2017).

*TNXB* contributes to angiogenesis and the decrease of TGFβ1 expression level in *TNXB* knockout mice was reported as modulated by VEGF-A. *TNXB* acts by encouraging neovascularization and by suppressing anti-angiogenic VEGF-B mRNA expression *in vivo* (Sumioka et al., 2018). Moreover, it is mutated in a small cohort of patients affected by Ehlers-Danlos syndromes, a heterogeneous group of rare monogenic conditions characterized by vascular and generalized connective tissue fragility (Malfait, 2018).

*AOC3* encodes for the amino oxidase enzyme also expressed in cerebral venules and in blood-brain barrier capillaries (https://discovery.lifemapsc.com/in-vivo-development/brain/blood-brain-barrier/endothelial-cells). Associated phenotypes include different vascular impairments (Vijayan and Reddy, 2016).

*FLRT3* encodes for the fibronectin leucine rich transmembrane protein 3, an extracellular adhesion molecule, implicated in several cellular processes as axon guidance, pericardium, and retinal vasculature development.

Based on current knowledge, the other loci here considered are not involved in angiogenesis or in vascular differentiation.

However, among all selected LGD variants, we found the *de novo* c.569G > A (p.Trp190Ter) nonsense mutation affecting *STK4* gene that leads to a truncated protein and the our hypothesis is that this variant could mainly be involved in the development of the bAVM phenotype. This is due to different reasons. Firstly, it is a *de novo* mutation harbored only by the young patient and this could be justify the onset of the sporadic condition.

Homozygous mutations in *STK4* are linked to the autosomal recessive primary immunodeficiency (Abdollahpour et al., 2012). The nonsense mutation here we reported was detected in heterozygous condition and this can be well accepted if we consider the dominant pattern of inheritance of bAVM. Moreover, RT-PCR and western blot data showed a statistically significant reduction both of the *STK4* transcript and of the protein in mutated sample, when compared to the wild-type one. Gene Ontology annotations for biological processes report *STK4* involved in “branching involved in blood vessel morphogenesis” (GO:0001569), defined as the process of coordinated growth and sprouting of blood vessels giving rise to the organized vascular system. In particular, recent evidence suggests that hypoxia-induced reactive oxygen species (ROS) biosynthesis from mitochondria could be a major upstream regulator of Stk4 activation in endothelial cells. Activated stk4 promotes planar and perpendicular vascular branching for regulating tip endothelial cells polarity and sprouting angiogenesis (Kim et al., 2019). Moreover, it was recently shown that *STK4* acts as pro-apoptotic factor in damaged venous endothelial cells by the MIEF1 pathway. In particular, the study was conducted on human umbilical vein endothelial cells (HUVECs) treated with tumor necrosis factor α, in order to mimicking lower limb chronic venous disorders. Chronic venous disorders, indeed, also include telangiectasias (Lu et al., 2019). *STK4* was firstly identified as a member of Hippo signal pathway that regulates cell proliferation, growth, differentiation, and apoptosis (Varelas and Wrana, 2012). A crosstalk between the Hippo pathway and the BMP/TGFβ signaling was demonstrated in endothelial cells and this finding could elucidate a potential role of Hippo pathway in bAVM and HHT pathogenesis (Young et al., 2015).

In addition, it is well known that human brain AVMs over-express COUP-TFII, PROX1, and other genes, also known to regulate key aspects of angiogenesis and lymphangiogenesis. These genes have similar expression patterns and a subset of them signiﬁcantly correlated with the presence of preoperative edema and acute hemorrhage (Shoemakerl et al., 2014). *STK4* is not included among these genes, but homozygous nonsense mutation in *STK4* cause primary immunodeficiency syndrome, given its central role in immunity (Abdollahpour et al., 2012).

Based on these evidence, we think that consequences on angiogenesis of this loss of function mutation must be further validated in order to confirm our hypothesis of its role in the development of bAVM.

However, also a pathogenic model based on interaction among low-penetrance loci could be considered. In this context, the other genes we selected could act as modifier genes. This idea comes from the observation of their expression profile. In particular, TGFβ, BMP, and IGF family members are highly expressed at embryo stage particularly in mesoderm, by which originate vasculature, connective tissue, skeleton, and urinary tract. All considered variants affect genes associated to connective tissue, glomerulus, and skeleton impairment (Demirdas et al., 2017; Emlet et al., 2017; Schock et al., 2017; Wunnenburger et al., 2017). However, a critical point regards the clinical significance of variants detected by the analysis. Although some of these have already been reported in variant collection databases, no information is available about the phenotypes of the variant carriers. In this context, a valid example is given by rs147182183 (c.1204G > C, p.Ala402Pro) affecting *BMP3* locus: it is reported in SNP collection databases with the very low allele frequency equal to 9.127e−05 in European non-Finnish population. However, it is unknown if it was found in healthy subjects or in affected patients, also asymptomatic. *BMP3*, indeed, is a relevant candidate locus. Moreover, high variant rate in genes involved in BMP/TGF-β and VEGF/VEGFR pathways was detected in a cohort of 100 patients affected by sporadic bAVM (Wang et al., 2018).

Among selected LGD variants and according to literature data, clearly *NBPF10*, *EFNA4*, *NAXE*, *TTC21B*, *BMP3*, *IGFBP7*, *SLIT2*, *CD109*, *TNXB*, *CSPG4*, *AOC3*, *NEDD4L*, *STK4*, and *FLRT3* could be valid candidate loci to consider in a more wide cohort of patients. In particular, *STK4* resulted affected by a *de novo* loss of function mutation. Limits of this study are indeed the single-case considered and the lack of functional data about the effects of these variants on vascular development. Genotyping of a larger cohort of patients will allow to more finely select mutations or genes for functional validations.

## Data Availability Statement

The raw data supporting the conclusions of this article will be made available by the authors, without undue reservation, to any qualified researcher.

## Ethics Statement

The study was approved by the local Ethical Committee A.O.U. “G. Martino”, Messina. The study involves human participants and was performed according to Helsinki declaration. All involved subjects were fully informed and written consent was obtained.

## Author Contributions

CS wrote the manuscript and conducted laboratory investigation. LD performed bioinformatic analysis. CA and FG recruited the patient and formulated the diagnosis. CR contributed to laboratory investigation. ML provided at financial support. RD’A conceived the study and corrected the manuscript. AS coordinated the research team.

## Conflict of Interest

The authors declare that the research was conducted in the absence of any commercial or financial relationships that could be construed as a potential conflict of interest.

## Supplementary Material

Supplementary Table 1 The table lists the nodes represented in Figure 2A. For each node are reported the node IDs in the different databases (Ensembl, Entrez, RefSeq. UniProt). Node type refers to the origin of the node within the network: query nodes were given as input while result nodes were inferred by the analysis.

Supplementary Table 2 The table explains the details of the network edges reported in Figure 2A. For each edge, the nature of the link (co-expression, co-localization, genetic interaction, shared protein domains) and the source (network column) are also provided. The other columns show the statistical parameters derived from network assembly.

Supplementary Table 3 Edge table of Figure 2B. For each edge, the nature of the link (co-expression, co-localization, genetic interaction, shared protein domains) and the source (network column) are also provided. The other columns show the statistical parameters derived from network assembly.

Supplementary Table 4 Annotation table of nodes represented in Figure 2. Nodes are grouped based on GeneOntology annotation.
