# Supplementary material for: High-Throughput Sequencing to Detect Novel Likely Gene-Disrupting Variants in Pathogenesis of Sporadic Brain Arteriovenous Malformations
Source: Front Genet. 2020 Feb 28;11:146. doi: 10.3389/fgene.2020.00146 (PMC7059193; doi:10.3389/fgene.2020.00146)
Supplement: Supplementary file 2 [file DataSheet_1.pdf]

SM1: Primer sequences and PCR conditions

Gene	Cds position	Primer forward	Primer reverse	Fragment length (bp)	T.A. (°C)
NBPF10	c.1308-4G>A (rs4110417)	5'-GTGGGAAATACCTGAACGAA-3'	3'-AGGGTGTGCCTCCTAGATAT-5'	178	56
EFNA4	c.103A>G*	5'-ATTGGCGGAAAGTTATTCAGG-3'	3'-CACGCTCTTTCATAAGAGTAA-5'	657	59
ABL2	c.2789A>G (rs17277288)	5'- AGAGGAAAGTGCTGCTCCAA-3'	3'-GATGCTGCAGTAGTATCACT-5'	473	59
NAXE	c.23T>C*	5'-AGCACTTATTATCAAAGGGCC-3'	3'-GCTGAACTGGTATTCGTTAAAT-5'	633	55
TTC21B	c.179T>A (rs371571631)	5'-TGGAAGTGATCCAGTCTTCA-3'	3'-TGAAACCTCTGCCAGAGGAT-5'	478	56
CLCN2	c.739G>C*	5'-TTGTGCATATCGCAAGCATGT-3'	3'-GGCTGTAATAGTCTCTAAAGG-5'	597	55
BMP3	c.1204G>C (rs147182183)	5'-ACGCTCCAATTTGATGAGCA-3'	3'-CCAATACTGACTTCATATGAC-5'	407	60
IGFBP7	c.506T>C*	5'-AAATTTGCTTCAGATAATCCATC-3'	3'-TTTGTTAGCAGCATCCTAGAAT-5'	444	55
SLIT2	c.139C>T*	5'-AGTTCATCCTTGGGAGACAG-3'	3'-AGAGAACTAGCCAGCAAGGA-5'	774	55
CD109	c.1709C>T (rs41266745)	5'-ACTGTGAGAAGTTCTATGAC-3'	3'-TTGACTTACACATGCAAAGG-5'	479	55
TNXB	c.6286C>G*	5'-AAGAGTAGGCCTCTCTGAACT-3'	3'-CTGTCCTGAGCCTTTAGTGAA-5'	568	57
NCF1	c.269G>A (rs201802880)	5'-TAGGTCACTTCCAATCTCGT-3'	3'-CTGAGGTGTGAAAGATTAAG-5'	619	53
TRMT10B	c.200G>T*	5'-TTCTCTAAACACGATCCAGTC-3'	3'-TCCTACTCTCATTGGAACATG-5'	350	55
TTLL11	c.1985G>T*	5'-GTGTTATATCATTAATTCTTTG-3'	3'-CCATCATATTCATTAATTCAC-5'	479	52
L2HGDH	c.718A>T*	5'-ATAGCTATGTCTATATTGGAGG-3'	3'-CAGAGCCACTTAATTAGGACA-5'	507	55
CSPG4	c.3239G>A (rs374794981)	5'- GTGACATCCTTCACCAATGAA-3'	3'-TAGGGCAATGGTCACTTGTA-5'	828	59
AOC3	c.1084G>A*	5'-CTATCCAGAAGGTGTTCTATC-3'	3'-CTGGTTCTGTTCAAACACACA-5'	530	55
NEDD4L	c.2488-7C>T (rs746481322)	5'-ACTACTTGAATCTTCTAGAT-3'	3'-TCATCTAAAGTTACTCTCTA-5'	398	48
STK4	c.569G>A*	5'-TCTTCTATGCCTGCTTGATC-3'	3'-TCAAGTGATTCTCATGCCTC-5'	634	62
FLRT3	c.1943A>T*	5'-CTCAAGGAACTGTGCATATAG-3'	3'-TACATCAATCGCAGCAGTAAC-5'	472	55

Table 1 SM: primers for PCR and Sanger validation. *novel variant.

PCR reaction mix

5X Q5 Reaction Buffer: final concentration 1X

10 mM dNTPs: final concentration 200 µM

10 µM forward primer: final concentration 0.5 µM

10 µM reverse primer: final concentration 0.5 µM

Template DNA: < 1 ng

Q5 High-Fidelity DNA Polymerase: final concentration 0.02 U/µl

*5X Q5 High GC Enhancer: final concentration 1X

Nuclease-Free Water: up to 50 µl

*supplied for NBPF10, TTC21B and CSPG4 amplicons

Thermocycling Condition

Initial denaturation: 98°C - 30 seconds

35 cycles: 98°C – 5 seconds

T.A. – 15 seconds

72°C – 10 seconds

Final extension: 72°C – 2 minutes

SM1: RFLP condition for allele frequency estimation

Gene	Variant	Amplicon length (bp)	Restriction enzyme	Digestion condition	Digested allele	Restriction fragments length (bp)
EFNA4	c.103A>G	657	BstEII	60°C, overnight	Mutated	254 + 403
CLCN2	c.739G>C	597	BtgI	37°C overnight	Wild-type	192 + 405
IGFBP7	c.506T>C	444	BstNI	60°C, overnight	Wild-type	178+208+58
TNXB	c.6286C>G	568	HaeII	37°C, overnight	Mutated	182 + 386
L2HGDH	c.718A>T	507	MfeI	37°C, overnight	Wild-type	284 + 223
SLIT2	c.139G>A	774	BsgI	37°C, overnight	Mutated	487 + 287

RFLP condition for allele frequency estimation. For each variant (2nd column) are reported the affected locus (1st column), the length of the amplicon to digest (3rd column), the restriction enzyme and the digestion conditions (4th and 5th columns), the digested allele and the length of fragments produced after digestion (6th and 7th columns).
