# Supplementary material for: High-Throughput Sequencing to Detect Novel Likely Gene-Disrupting Variants in Pathogenesis of Sporadic Brain Arteriovenous Malformations
Source: Front Genet. 2020 Feb 28;11:146. doi: 10.3389/fgene.2020.00146 (PMC7059193; doi:10.3389/fgene.2020.00146)
Supplement: Supplementary file 3 [file DataSheet_2.pdf]

SM2: Reports of WES analysis

Parameter	Value
**Output report**	
Total reads	86,816,620
Total yield (Gbp)	8.77
Read length (bp)	101 x 2
Q20	97.22%
Q30	92.83%
Read length	101 x 2
Coverage uniformity (10x)	≥ 90%
**Mapping**	
Deduplicated read (%), of these	69,592,810 (80.47)
Mapping read (%)	68,958,221 (99.09)
Unique read (%)	64,968,270 (93.35)
On-target read (%)	57,705,760 (82.92)
**Coverage distribution**	
Raw sequence depth	145.04
On-target depth	78.62
Coverage 20x rate	97.03
Coverage 50x rate	70.5

WES analysis report. Output data. Total reads: number of generated reads; total yield (Gbp): Giga base pairs of produced sequence data; Q20: quality ratio satisfying Phred quality score greater than 20, which represents an error rate of 1 in 100, with a corresponding call accuracy of 99%; Q30: quality ratio satisfying Phred quality score greater than 30, which represents an error rate of 1 in 1000, with a corresponding call accuracy of 99.9%; read length: length of paired-end reads generated. **Mapping report.** Deduplicated read: discarded clean reads following PCR duplicate; mapping read: de-duplicated reads followed by mapping onto the reference genome; unique read: reads with the same starting position on each end; on-target read: mapped de-duplicated reads (on-target region).

Coverage distribution. Raw sequencing depth: depth of raw data generated by sequence run; on-target depth: depth of on-target reads; coverage 20X rate (%): the rate of cumulative mapping depth against reference genome coverage more than 20X; coverage 50X rate (%): the rate of cumulative mapping depth against reference genome coverage more than 50X.

**Annotated variants categories**	**Number of detected variants**
Regulatory region	
UTR regions	32,228
Intergenic	2,231
Splice site region	1,586
Non-coding RNA targeted	4,095
Exon	
Non-synonymous	11,191
Nonsense	87
Synonymous	11,244
Frameshift	272
Inframe ins/dels	203
No-start	21
No-stop	14

Variant calling and annotation results. Number of annotated variants for each functional class.
